# A81 GASTROINTESTINAL ADVERSE EVENTS ASSOCIATED WITH GLUCAGON-LIKE PEPTIDE-1 (GLP-1) AGONISTS IN OVERWEIGHT PATIENTS: A SYSTEMATIC REVIEW AND META-ANALYSIS

**DOI:** 10.1093/jcag/gwae059.081

**Published:** 2025-02-10

**Authors:** Y Verma, N Gimpaya, W Tran, R Sondawle, G Sinanian, D Tham, S Grover

**Affiliations:** University of Toronto Temerty Faculty of Medicine, Toronto, ON, Canada; Scarborough Health Network, Scarborough, ON, Canada; Education Research, Scarborough Health Network, Scarborough, ON, Canada; Education Research, Scarborough Health Network, Scarborough, ON, Canada; Scarborough Health Network, Scarborough, ON, Canada; Scarborough Health Network, Scarborough, ON, Canada; Scarborough Health Network, Scarborough, ON, Canada

## Abstract

**Background:**

Glucagon-Like Peptide-1 (GLP-1) agonists are widely prescribed for managing type 2 diabetes and have recently gained attention for their potential in promoting weight loss in obese patients. The off-label use of certain GLP-1 agonists for weight loss has been steadily increasing, raising concerns about their safety in non-diabetic patients. Although the gastrointestinal (GI) side effects of GLP-1 agonists are well-documented in diabetic populations, their impact on overweight or obese individuals has not been systematically analyzed.

**Aims:**

To systematically review and analyze the incidence, severity, and types of GI adverse events (AEs) associated with the use of GLP-1 agonists in overweight or obese patients (BMI: >25 kg/m^2^)

**Methods:**

We conducted a systematic search across five databases, including PubMed, Ovid MEDLINE, Embase, Web of Science, and Scopus. Studies were restricted to randomized controlled trials (RCTs) involving GLP-1 agonists whose primary indication was obesity and whose primary outcome was weight loss. Studies that did not report adverse events were excluded. Adverse events were extracted using their preferred terms from the Medical Dictionary for Regulatory Activities (MedDRA). A meta-analysis was performed to pool the relative risk (RR) of GI AEs using the random effects model. A meta-regression was also performed.

**Results:**

A total of 42 studies (n = 23318 patients) met the inclusion criteria. GLP-1 agonists showed an increased risk of nausea (RR 2.77; 95% CI 2.50–3.10; p < 0.001), diarrhea (RR 2.01; 95% CI 1.78–2.01; p < 0.001), and constipation (RR 2.13; 95% CI 1.90–2.38; p = 0.05) when compared to placebo. The incidence of nausea was higher in those with lower average BMI (coefficient: 3.88; p = 0.005). Additionally, we found no difference between GLP-1 agonists and placebo in the risk of cholelithiasis, cholecystitis, and biliary dyskinesia (RR 1.62; 95% CI 1.22–2.17; p = 0.99).

**Conclusions:**

Our study found that obese patients are at an increased risk of experiencing nausea, diarrhea and constipation when using GLP-1 agonists compared to placebo. Gallbladder disorders showed no difference in risk. For obese patients, lower average BMI may increase the likelihood of experiencing nausea.

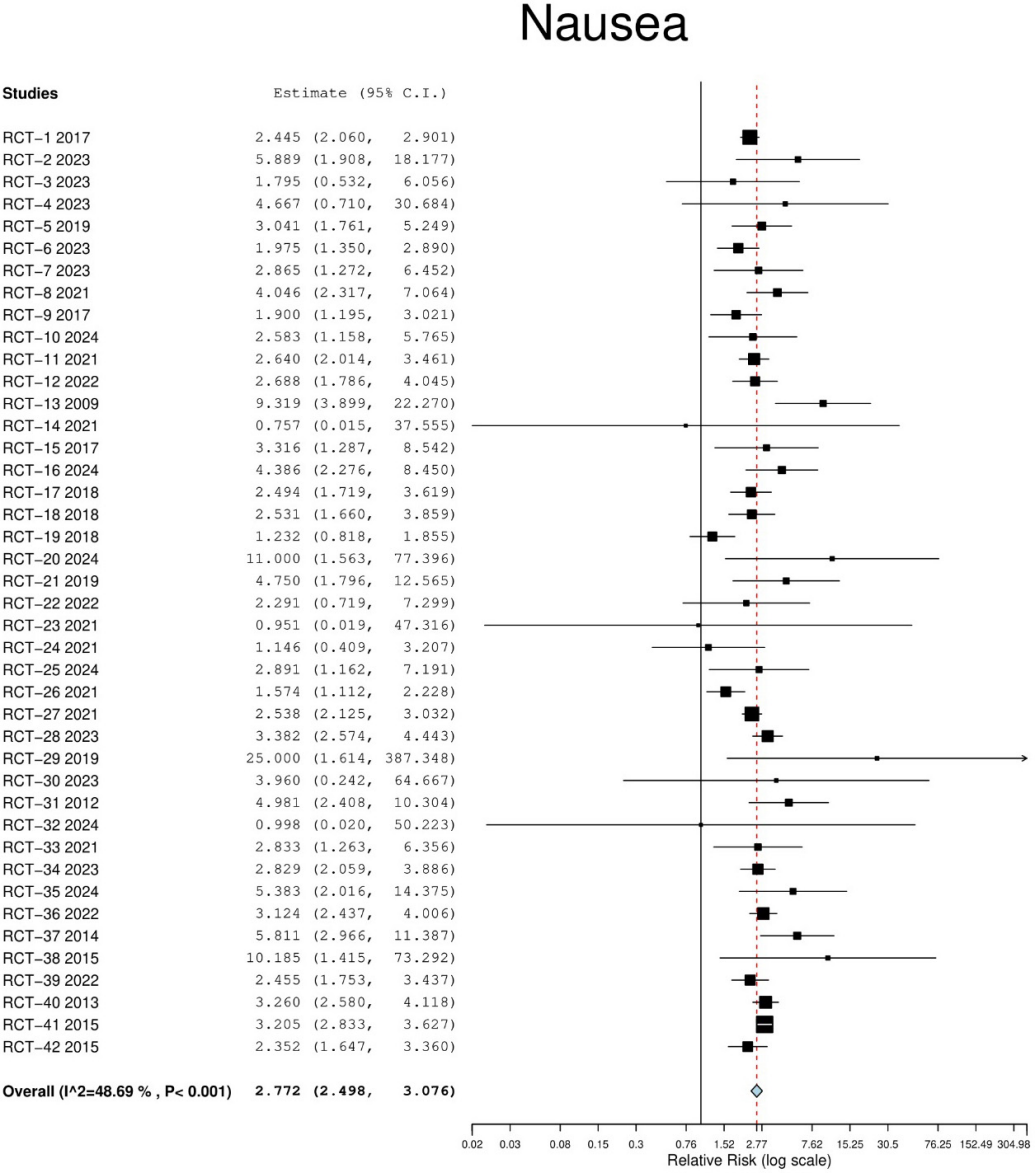

FIgure 1. Forest plot indicating relative risk for nausea in obese patients treated with GLP-1 agonists. Error bars represent 95% confidence intervals.

**Funding Agencies:**

None

